# Intimate partner violence against women: Operationalized Psychodynamic Diagnosis (OPD-2)

**DOI:** 10.1371/journal.pone.0239708

**Published:** 2020-10-01

**Authors:** Luciane Maria Both, Taís Cristina Favaretto, Lúcia Helena Machado Freitas, Sílvia Pereira da Cruz Benetti, Carla Crempien

**Affiliations:** 1 Psychiatry and Behavioral Sciences, Federal University at Rio Grande do Sul, Porto Alegre, Brazil; 2 Department of Psychiatry and Legal Medicine, Federal University of Rio Grande do Sul and Psychiatric Service, Hospital de Clínicas de Porto Alegre, Porto Alegre, Brazil; 3 Center for Studies and Treatment of Psychic Trauma, Psychiatric Service, Hospital de Clínicas de Porto Alegre, Porto Alegre, Brazil; 4 The University of Vale do Rio dos Sinos, São Leopoldo, Brazil; 5 Millenium Institute for Research in Depression and Personality, Santiago, Chile; 6 Pontificia Universidad Católica de Chile Diplomado Diagnóstico, Indicación y Estrategias en Psicoterapia: Diagnóstico Psicodinámico Operacionalizado (OPD-2), Santiago, Chile; University of Sao Paulo Medical School, BRAZIL

## Abstract

**Introduction:**

Intimate partner violence against women is one of the most common forms of violence. Different research fields are trying to understand the cycle of violence, such as the psychological field, to understand how these women's relational patterns and intrapsychic conflict function in the cycle of violence.

**Objective:**

To investigate the operationalized psychodynamic diagnosis of women victims of domestic violence, exploring the severity and experience of violence, structural functions, dysfunctional interpersonal patterns, and intrapsychic conflicts.

**Method:**

We conducted a cross-sectional quantitative study using the OPD-2 Clinical Interviews, which were recorded and transcribed. The sample was composed by 56 women victims of domestic violence, mean age 30.07 (SD = ±9.65). Reliability was satisfactory for judges interviews(k>0,6).

**Results:**

According to the OPD-2 evaluation, we found that the severity of the violence was associated with the intensity of women's subjective suffering. In the relational pattern, they stay in the relationship, leaving themselves vulnerable; perceive the partner as controlling, aggressive, offensive, and fear abandonment. As a defensive mechanism to relational discomfort and suffering victims anticipate the aggressor's desire, resulting in submissive behavior. The main psychic conflict was the "need for care versus self-sufficiency" (78.6%). And medium was the predominant structure level, in which they presented insecure internal objects, presenting difficulties in emotional regulation and perceiving reality in a distorted way. Hence, they do not recognize their limitations and needs. We found that 78.6% of the cases had some psychiatric disorder: MDD, PTSD.

**Conclusion:**

This study provides empirical evidence on clinical observations on the psychological functioning of this population and the issues that make up the maintenance of domestic violence against women. The understanding of internalized patterns, structural functions, and motivational tensions are fundamental for the prevention of re-victimization and improving coping mechanisms, as well as promoting greater adherence to treatment.

## Introduction

Intimate partner violence (IPV) against women, often called domestic violence, is one of the most common forms of violence. Refers to any behavior by an intimate partner or ex-partner that causes physical, sexual or emotional abuse, including physical aggression, sexual coercion, psychological abuse, and controlling behaviors, where one partner exercises power and control over the other. It occurs in all settings and among all socioeconomic, religious, and cultural contexts. Women bear the overwhelming global burden of IPV [1]. It is a human rights violation with significant health consequences [2], like mental health problems, sexual and reproductive problems, and chronic conditions—and represent a significant health burden for women [3].

There is a substantial body of literature documenting the prevalence of violence against women. International evidence suggests that domestic violence against women constitutes a global public health problem. Male partners commit approximately 38% of all murders of women [1]. More specifically, 23% of women suffer violence from their partners [4].

Data from the latest Institute for Applied Economic Research (*Instituto de Pesquisa Econômica Aplicada*–IPEA) report on the Atlas of Violence [5], which provides information on the number of femicides, show that in 2016 alone, 4,645 women were murdered in Brazil, corresponding to 4.5 deaths per 100,000 women. And data from 2017 indicate that the number of femicides in absolute terms in Brazil, with 1,133 victims confirmed in 2017 [4].

The dynamics of victimization through abusive relationships imply repetitive behavioral patterns, maintaining the cycle of violence. Usually it presents itself with a slow and silent beginning followed by gradual progress towards acts of humiliating beatings, physical violence, public manifestations of aggression. Moreover, to make matters worse, some victims' responses to coping with constant threats such as shame and isolation reinforce the cycle of violence. These women have difficulty in reporting violence and accessing legal information. In general, care services are of low quality and cannot protect victims [6].

There is strong evidence supporting the possibility of reducing the indices through effective prevention strategies [7]. So, understanding the victim’s perspective is essential to developing policy and practice standards, as well as instructing professionals working in the justice system and policy-making. It is necessary to understand the psychodynamic mechanisms underlying these women's behaviors, from the Operationalized Psychodynamic Diagnosis [8]. Many women remain and repeat abusive relationships, but the motives behind these are unclear. Thus, this study aims to investigate the operationalized psychodynamic diagnosis of women victims of domestic violence, exploring the severity and experience of violence, structural functions, dysfunctional interpersonal patterns, and intrapsychic conflicts.

## Hypotheses

We expect to find a dysfunctional interpersonal functioning with a history of violence or family neglect in childhood (transgenerationality), which ultimately creates a bond of dependency, and the partner abuses this power. These aspects are associated with conflicts, Axis III, based on the predominance of conflicts: autonomy x dependency, the need for care x self-sufficiency, and submission x control. However, it also indicates the conflict of self-esteem, guilt, oedipal, and identity.

Regarding Axis I, we hypothesize intense suffering, few resources available due to PTSD, and resistance to change in family dynamics, and maintenance of violence. In Axis II, we predict interpersonal relationships permeated by abandonment, devaluation, censorship. In Axis IV, we expect to find a more disintegrated structure, emotional dysregulation, emotional inflexibility, negative internal representations, and non-self-object differentiation. And in Axis V, we believe that there will be PTSD with comorbidities such as major depressive disorder, generalized anxiety, among other problems. We expect to find sociodemographic variables, low income, and low educational level of the victims.

## Methods

### Study design

This is a quantitative cross-sectional study, developed from the Diagnostic Psychodynamic Operationalized (OPD-2). It is part of a larger study still in progress about IPV in general.

### Sample

Fifty-six women victims of domestic violence from a specialized public service in southern Brazil participated in the study. We included women 18 to 65 years of age who were victims of self-reported domestic violence who sought the service along the data collection period during the on-call researcher's shift.

The sample calculation used was based on the validation study by Krieger [9]. Porto Alegre Clinical Hospital (*Hospital de Clínicas de Porto Alegre*) calculated the sample size, where we considered the number of items of the OPD instrument and the literature data on the agreement between evaluating judges for the different axes of the instrument. Thus, the number of interviews was calculated for each axis independently: Axis 1: 53 interviews; Axis 2: 52 interviews; Axis 3: 53 interviews; Axis 4: 25 interviews. Thus, to compose the sample of this study, a minimum of 53 participants.

### Instrument

We applied the sociodemographic data survey regarding characteristics relevant to the study, such as age, educational, family income, the period of marital relationship, living situation, the use of drugs, the existence of parental violence (transgenerationality of violence), and so forth. We used the sociodemographic questionnaire based on the study developed by Lourenço and Baptista [10].

The Operationalized Psychodynamic Diagnosis (OPD-2) was used to operationalize psychodynamic constructs, to formulate a multiaxial psychodynamic diagnosis, and for therapeutic planning and focus. The OPD-2 is composed of five axes. In Axis I, we evaluated therapeutic indication and motivation, then the diagnosis is made. In Axis II—Relational, in the manifestation of conflicts or vulnerabilities of the interpersonal representations -, Axis III—Conflict—and Axis IV—Structural vulnerabilities and capacities. And Axis V—Nosological diagnosis [8]. However, the adaptation of Axis I, "Module for the Evaluation of Domestic Violence OPD" proposed by the Chilean researcher Carla Crempien in 2009 in her Ph.D. thesis [11, 12] and adapted to Brazil [13].

We used the OPD Clinical Interview, a semi-structured interview with specific interviewing tools for the analysis of each axis, described in the manual on pages 498 to 524. For coding, two expert judges trained in the use of the OPD system scored each item according to the criteria specified in the OPD-2 Manual [8].

The Chilean validation of the OPD-2 showed a significant agreement between evaluating judges: 75% in axis II, 73.3% in axis III, 62% in axis I, and 53.3% in axis IV [14]. In Portugal and Brazil: 78% in axis IV, 66% in axis I, 57.7% in axis III, and axis II was excluded [15]. Particularly in Brazil, according to Krieger [9], there are adequate psychometric properties for the applicability of OPD-2 in the Brazilian population.

Authorization for the use of Operationalized Psychodynamic Diagnosis in Brazil was requested from the president of the OPD Group, Dr. Manfred Cierpka and Carla Crempien who carried out the adaptation of Axis I to assess women who suffered domestic violence.

### Procedures for collecting and setting

We invited the victims of violence to participate voluntarily in the research during the specialized service during the months of November and December 2017. While waiting for their screening, the victims answered the sociodemographic data sheet. Later, in the specialist's room, the researcher conducted the OPD Clinical Interview and provided information on safety procedures and the rights guaranteed by the Maria da Penha Law for victims [16]. Each interview lasted approximately one hour [14]. Only one researcher did the interviews. These interviews were audio-recorded and transcribed. Also, using the sociodemographic datasheet to characterize the sample.

### Data analysis

Firstly, data analysis was done by two independent trained judges who coded the interviews in the OPD worksheet. The evaluating judge codified the various psychodynamic aspects from the dimensions and indicators described in the manual that integrate the scores: 0 (absent), 1 (mild/insignificant), 2 (moderate), 3 (high/significant), 4 (very significant), 9 (non-assessable). All the evaluating judges completed specific training in the instrument: "Diploma in Diagnosis, Indication, and Strategies in Psychotherapy: Operationalized Psychodynamic Diagnosis (OPD-2)", organized by the School of Medicine of Pontificia Universidad Católica de Chile.

The kappa coefficient of each axis was calculated independently for each interview. Later, analyses conducted by SPSS software. We considered *p*<0,05 to define statistical significance. In this study, concordance between the judges was substantial in each axis; 63% in Axis I violence module, 73% in Axis III, 82% in Axis IV, and 100% in Axis V. For Axis II, we considered the items scored more frequently by judges.

### Ethics statement

This study was approved by the Federal University at Rio Grande do Sul ethics committee (CAAE 68271917.7.0000.5347, No. 2,412,749) and permission for collection of data was obtained from the Legal Medical Department of Porto Alegre. All participants were invited voluntarily and authorized their participation in the research by signing the Term of Free and Informed Consent. All data that could identify participants was omitted.

## Results

### Study population

The sample had a normal distribution, according to the Kolmogorov-Smirnov normality test (*p* <0.05). The mean age of women was 30.07 (SD = ± 9.65) years, and men presented a mean of 34.8 (SD = ± 10.86) years (Table 1). In the sample, 46,4% ended their relationship in less than six months (Table 2).

**Table 1:** Sociodemographic data.

Category	Subcategory	Women	Men
Age	18 to 20 years old	8 (14,3%)	3 (5,4%)
	21 to 25 years old	16 (28,6%)	5 (8,9%)
	26 to 30 years old	8 (14,3%)	16 (28,6%)
	31 to 35 years old	9 (16,1%)	13 (23,2%)
	36 to 40 years old	8 (14,3%)	4 (7,1%)
	41 to 45 years old	2 (3,6%)	5 (8,9%)
	46 to 50 years old	3 (5,4%)	5 (8,9%)
	51 to 55 years old	1 (1,8%)	1 (1,8%)
	56 to 60 years old	1 (1,8%)	2 (3,6%)
	61 to 65 years old	0	2 (3,6%)
Race	White	37 (66,1%)	35 (62,5%)
	Black	21 (21,4%)	13 (23,3%)
	Brown	6 (10,7%)	7 (12,5%)
	Indigenous	1 (1,8%)	1 (1,8%)
Scholarity	Illiterate	0	1 (1,8%)
	Incomplete elementary school	15 (26,8%)	17 (30,4%)
	Complete primary education	6 (10,7%)	6 (10,7%)
	Incomplete high school	8 (14,3%)	7 (12,5%)
	Complete high school	22 (39,3%)	22 (39,3%)
	Incomplete higher education	3 (5,4%)	1 (1,8%)
	Complete higher education	1 (1,8%)	2 (3,6%)
	Postgraduate studies	1 (1,8%)	0
Income^a^	None	13 (23,2%)	8 (14,3%)
	Less than 1 salary	8 (14,3%)	1 (1,8%)
	Between 1 and 2 salaries	31 (55,4%)	35 (62,5%)
	Between 3 and 6 salaries	2 (3,6%)	5 (8,9%)
	Between 7 and 12 salaries	1 (1,8%)	2 (3,6%)
	More than 12 salaries	1 (1,8%)	4 (7,1%)
Religion	Godless^b^	22 (39,3%)	26 (46,4%)
	Catholic	19 (33,9%)	15 (26,8%)
	Spiritism^c^	4 (7,1%)	3 (5,4%)
	Afro-Brazilian	3 (5,4%)	6 (10,7%)
	Evangelical	0	6 (10,7%)
Addiction	Alcohol	3 (5,4%)	25 (44,6%)
	Drug	2 (3,6%)	0
	Tobacco	8 (14,3%)	7 (12,5%)
	Marijuana	1 (1,8%)	7 (12,5%)
	Cocaine	0	3 (5,4%)
	Marijuana and Cocaine	0	7 (12,5%)
	Anabolic	0	1 (1,8%)

^a^One salary is a basic remuneration to the worker.

^b^Godless is the person who do not believe in God.

^c^Spiritism is the person who believe in life after death.

**Table 2:** Relationship.

Category	Subcategory	Women
Type of relationship	Date	1 (1,8%)
	Courtship	9 (16,1%)
	Marriage	5 (8,9%)
	Stable union	8 (14,3%)
	Divorced	7 (12,5%)
	Separated in less than 6 months	26 (46,4%)
Time of relationship	Less than 6 months	3 (5,4%)
	Between 6 months and 1 year	6 (10,7%)
	Between 1 and 2 years	11 (19,6%)
	Between 3 and 5 years	13 (23,2%)
	Between 6 and 10 years	15 (26,8%)
	Between 11 and 15 years	2 (3,6)
	Between 16 and 20 years	4 (7,1%)
	Between 21 and 30 year	2 (3,6%)

Still, on the checklist questions with options to mark "yes" or "no" if the parents had a violent relational dynamic, 32 (57.1%) women reported not having witnessed discussions between their parents or suffered any situation of violence in childhood. Among the other women, 17.9% experienced daily violence between their parents. Despite this, most of them, 41 (73.2%), rated their father as loving and caring; and 43 (76.8%) evaluated mother's care in the same way. However, these data did not corroborate with the self-report part of the interview on situations in childhood, women described situations of violence in the parental dynamics. The univariate analysis did not indicate significant differences between the type of parental care and the psychodynamic dimensions.

### Main analysis

All women suffered both emotional and physical violence at relatively high intensities, corresponding to the severity of the violence. The victims' suffering was distressing, they reported very difficult situations, they mentioned things they didn't do out of fear, or because they felt unable to do so. During a lengthy interview, they complained and described the violent situations they experienced. They believed that violence occurred due to external factors, such as the husband's use of alcohol, in consequence of personal situations, or relational factors. Victims believe that external measures were necessary to overcome the cycle of violence, such as the protective measures provided by the protective organs in the court of law (Table 3). The first episode of violence was on average with 25.52 years of age, and 25% of the sample this violence occurred about 2–5 years ago (Table 4).

**Table 3:** Axis I—Mean and intensity of items assessed in the domestic violence module.

	Mean (SD)	Intensity^a^			
		1	2	3	4
**Type and severity of violence**					
Emotional violence	2,61(0,53)	0	23(41,1%)	32(57,1%)	1(1,8%)
Physic violence	2,38(0,62)	2(3,6%)	33(58,9%)	19(33,9%)	2(3,6%)
Sexual violence	0,23(0,81)	52(91,1%)	1(1,8%)	2(3,6%)	1(1,8%)
Global Severity Index	2,47(0,60)	1(1,8%)	30(53,6%)	23(41,1%)	2(3,6%)
**Subjective experience, presentation of the problem and personal concept**					
Intensity of subjective suffering	2,59(0,53)	0	24(42,9%)	31(55,4%)	1(1,8%)
Presentation of complaints on DV	2,39(0,53)	1(1,8%)	32(57,1%)	23(41,1%)	0
**Personal explanation of DV**					
Oriented to external causes	2,34(0,61)	3(5,4%)	32(57,1%)	20(35,7%)	1(1,8%)
Oriented to psychological/interpersonal causes	2,00(0,57)	9(16,1%)	38(67,9%)	9(16,1%)	0
**Change concept**					
Oriented to external modifications	2,43(0,53)	0	33(58,9%)	22(39,3%)	1(1,8%)
Oriented to personal changes	2,00(0,79)	17(30,4%)	22(39,3%)	17(30,4%)	0
**Personal resources and obstacles to change**					
Personal resources	1,91(0,61)	13(23,2%)	35(62,5%)	8(14,3%)	0
Personal obstacles	2,18(0,61)	6(10,7%)	34(60,7%)	16(28,6%)	0
External resources	2,05(0,44)	4(7,1%)	45(80,4%)	7(12,5%)	0
External obstacles	2,00(0,38)	4(7,1%)	48(85,7%)	4(7,1%)	0

^a^The higher the score is the higher the severity.

**Table 4:** Duration of violence.

Time	Frequency (%)
<6 months	18(32,1%)
6–24 months	8(14,3%)
2–5 years	14(25%)
5–10 years	6(10,7%)
>10 years	10(17,9%)

In the relational formulation of the patient—Axis II—was observed: 1. (how the victim sees others) Women repeatedly experience others as controlling, bossy, demanding, and aggressive. The other despise, belittle them, restrict their freedom, and neglect their needs. 2. (how the victim sees herself) Thus, given this attitude, the victims allow the aggressor to act autonomously and isolate themselves from other social activities. 3. (what she does but is unaware) The victims protect themselves in equally, allowing dangerous progression. They feel confused when the other shows affection, 4. (how the other reacts to victims’ unconscious proposal) Therefore, it induces an unconscious response to the other, who defies and imposes himself aggressively. The victim anticipates the aggressor's desire, as a defensive response to discomfort, and relational distress, therefore becomes submissive, thus keeping the relationship abusive. Sometimes, others (as well as the evaluator) noticed that repeatedly the patient, who cares and worries excessively about the aggressor, does not give him space and is intrusive, at the same time showing submissive attitudes and dependency.

**Fig 1:**
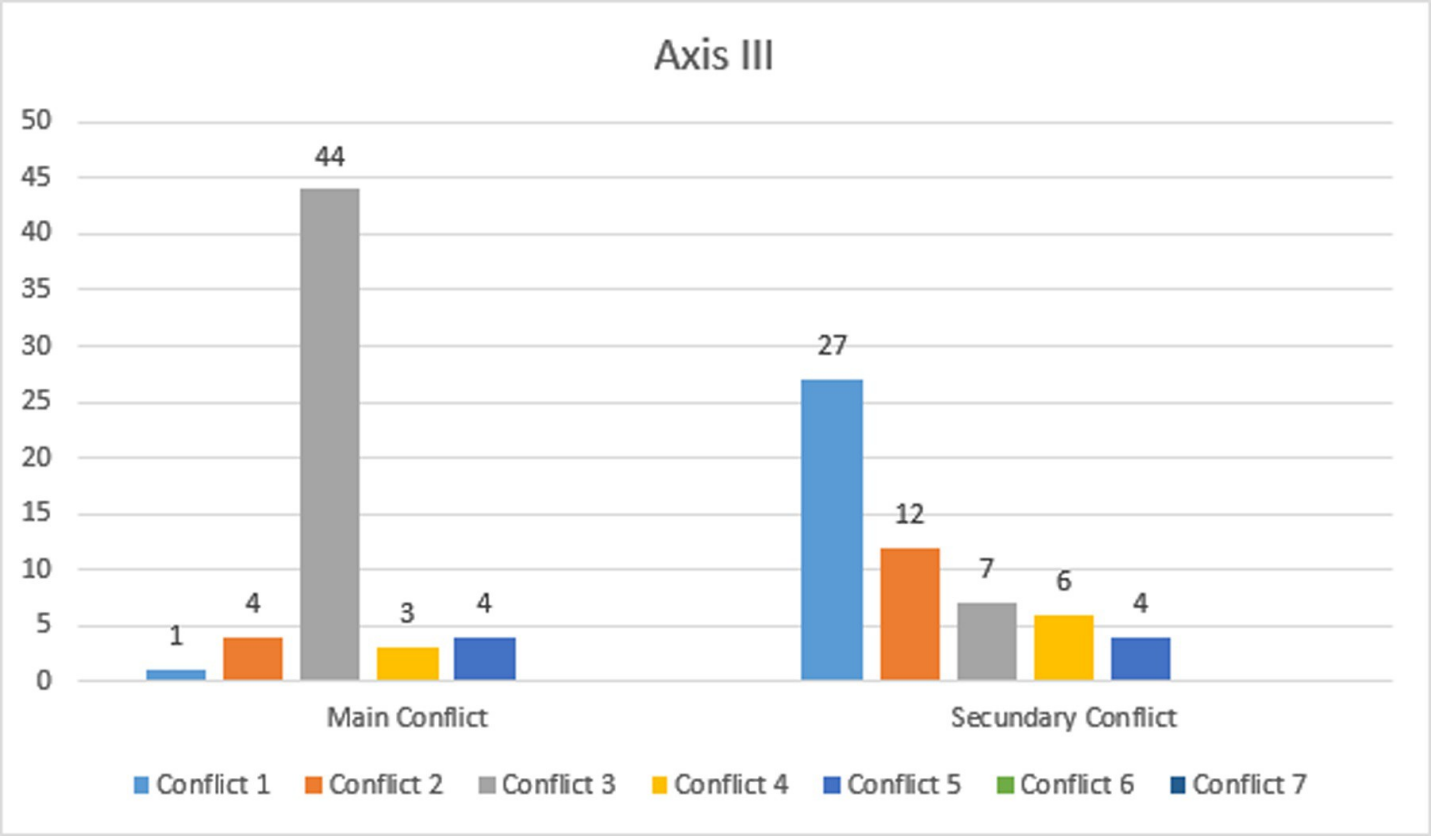
Axix III.

In the main and secondary conflicts, there was a significant difference between the frequency of the other conflicts (*p* <0.05)—Axis III. Thus, the main conflict identified was the "need for care versus self-sufficiency" (78.6%), and the second one was "individuation versus dependence" (48.2%), with a predominantly active mixed mode of actions (58.9%), as noted in Fig 1. The main conflict *"need for care versus self-reliance"* focuses on the fundamental needs of individuals to take or give in the exchange of security and care. Thus, they were very concerned about the other, whereas latent depressive feelings were a defense mechanism against the feeling of emptiness. They pretend to be self-sufficient, but the desire for care predominates. They desire for retribution for the dedication they gave.

We observed a level of median psychic structure—Axis IV, the reflexive perception of the reduced self. Introspection about one's present state was limited. Situations and events that generate tension weakened the coherence of self-image. The differentiation between what was personal and what was of the other is difficult. While questioning them, women did not describe themselves at a disadvantage in any situation. Situations and moods influenced them strongly, and they tried to remain stable by avoiding affection (Table 5).

**Table 5:** Axis IV–Structure.

	Minimum	Maximum	Mean^a^	SD
**Cognitive abilities**				
1a Self-perception	1,50	2,50	2,05	0,33
1b Object perception	1,50	2,50	2,14	0,31
**Regulation**				
2a Self regulation	1,50	2,50	2,16	0,25
2b Regulation of object relationship	1,50	2,50	2,21	0,27
**Emotional communication**				
3a Internal communication	1,50	2,50	2,07	0,23
3b Communication with the external world	1,50	2,50	2,08	0,23
**Attachment**				
4a Attachment to internal objects	1,50	3,00	2,23	0,32
4b Attachment to external objects	2,00	2,50	2,09	0,19
**5 Total structure**	1,50	2,50	2,10	0,22

^a^The closer to 1, the greater the structural integration. And closer to 4, greater disintegration.

In this study, Axis 5 is based on the diagnostic criteria of the Diagnostic and Statistical Manual of Mental Disorders (DSM-5). In 78.6% of cases had some psychiatric disorders. The majority presented Major Depressive Disorder (MDD, 51.8%), as the only diagnosis (41.1%) or associated with another psychopathology (10.7%). Furthermore, 23.3% of the participants corresponded to the diagnostic criteria of Post Traumatic or Acute Stress Disorder as the only diagnosis or 10.7% associated with MDD or Borderline. Finally, one of the participants intoxicated herself, attempting to numb her suffering (Table 6).

**Table 6:** Axis V.

Disorder	Frequency(%)
No disorder	12 (21,4%)
Major Depressive Disorder	23 (41,1%)
Post Traumatic Stress Disorder	10 (17,9%)
Acute Stress Disorder	3 (5,4%)
Intoxication by sedatives, hypnotics or anxiolytics without use disorder	1 (1,8%)
Major Depressive Disorder + Post Traumatic Stress Disorder	5 (8,9%)
Major Depressive Disorder + Tobacco-Related Disorder	1 (1,8%)
Acute Stress Disorder + Borderline Personality Disorder	1 (1,8%)

In the bivariate analysis with sociodemographic data, we verified that the age of the victim and the partner were factors associated with the presence of physical violence and period of the violence, whose intensity and duration increases with the age of both. There is a correlation between the educational level of women and the perception that the reasons for violence were not due to external factors, presenting more resources to deal with the situation of violence. Income was not a very significant factor in maintaining the cycle of violence (Table 7). However, for the subject's level of psychic structure and the victim's educational level was important in the capacity to regulate the relationship, whose abilities decrease with the increase of educational level. Income and relationship periods correlated negatively with emotional self-regulation, with lower income and shorter relationships, better self-regulation (Table 8).

**Table 7:** Bivariate analyzes: Correlation of demographic partner data with Axis I.

	Age	Scholarity	Income	Time of relationship	Partner's age	Partner's Scholarity	Partner's income	Emotional violence	Physical violence	Sexual violence	Global Severity Index	Duration of violence	Age 1 episode	Intensity of subjective suffering	Presentation of complaints on DV	Oriented to external causes	Oriented to psychological causes	Oriented to external modifications	Oriented to personal changes	Personal resources	Personal obstacles	External resources	External obstacles
Age	1																						
Scholarity	0,11	1																					
Income	,34*	,34*	1																				
Time of relat.	,21	-,13	,19	1																			
Partner's age	,60**	-,76	,61	,33*	1																		
Partner's sch.	-,14	,37**	,29*	,12	,72	1																	
Partner's inc.	,16	,54**	,25	,42	,15	,44**	1																
Emotional	,10	-,06	,01	,15	-,03	,02	-,01	1															
Physical	,39**	,01	-,22	,34	,31*	-,14	-,01	,24	1														
Sexual	-,15	-,26	-,10	,39**	-,04	,15	,09	,22	-,10	1													
Global sev. I.	,18	-,05	-,18	,30*	,10	,10	,28	,76**	,40**	,41**	1												
Duration	,44**	-,25	,15	,27*	,33*	-,23	-,24	,35**	,29**	-,06	,27*	1											
Age 1° ep.	,79**	,15	,20	,003	,44**	,03	,05	-,09	,23	-,21	,006	-,09	1										
Int. sub. suff.	,02	-,05	,29	,17	-,01	,22	,19	,52**	,15	,23	,49**	,25	,13	1									
Pres. comp.	,19	,03	,09	,05	,27*	,28*	,10	,30*	,32*	,12	,33*	,21	,11	,46**	1								
Ext. causes	,54	-,37**	-,16	,007	,07	-,09	-,38**	,08	-,28**	-,24	,06	,24	-,04	,21	,14	1							
Psic. causes	-,05	,17	,0	,03	-,08	,32*	,29*	,0	,0	,28*	,16	-,02	-,12	,36**	,36**	,0	1						
Orient. ext.	,13	,15	-,01	-,03	,05	-,17	-,08	,22	,22	,02	,27*	,20	-,05	-,01	,29*	-,01	,06	1					
Orient. pers.	-,26	,05	-,07	-,20	-,28*	,15	-,02	,18	-,11	,03	,08	,03	,32*	,39*	,35*	,19	,44**	,17	1				
Pers. recourses	,25	,63**	,27*	-,18	,07	,21	,31*	-,17	,04	-,22	-,18	-,19	,31*	-,17	-,06	-,31*	-,10	,12	,04	1			
Pers. obstacles	-,29*	-,24	-,40**	,12	,0	-,23	-,09	,22	,60	,14	,32*	,13	-,39*	,23	,17	,28*	,11	,15	,23	-,35**	1		
Ext. recourses	,26	,33*	,17	,03	,12	,05	,13	-,06	,19	-,39**	,16	,11	,18	-,06	,06	-,07	-,14	,21	,10	,62**	-,31*	1	
Ext. obstacles	-,03	-,13	-,18	-,12	-,04	-,16	-,04	,09	-,08	,0	,24	,03	-,02	,18	,18	-,08	,08	,09	,12	-,78	,32*	-0,11	1

**Table 8:** Bivariate analyzes: Correlation of socio demographic data with Axis IV.

	Age	Scholarity	Income	Time of relationship	Partner’s age	Partner’s scholarity	Partner’s income	1a Self-perception	1b Object perception	2a Self regulation	2b Regulation of object relationship	3a Internal communication	3b Communication with the external world	4a Attachment to internal objects	4b Attachment to external objects	5 Total structure
Age	1															
Scholarity	0,11	1														
Income	,34*	,34*	1													
Time of relationship	,21	-,13	,19	1												
Partner’s age	,60**	-,76	,61	,33*	1											
Partner’s scholarity	-,14	,37**	,29*	,12	,72	1										
Partner’s income	,16	,54**	,25	,42	,15	,44**	1									
1a Self-perception o	,03	-,26	-,21	,005	,008	-,07	-,26	1								
1b Object perception	-,13	-,23	-,20	-,06	-,05	,01	-,20	,55**	1							
2a Self regulation	-,23	-,04	-,28*	-,27*	-,23	-,04	,08	,39**	,39**	1						
2b Regulation of object relationship	-,01	-,31*	-,19	-,05	,27*	,08	-,05	,40**	,46**	,31*	1					
3a Internal communication	-,02	-,21	-,12	-,08	,02	-,06	-,05	,39**	,51**	,36**	,36**	1				
3b Communication with the external world	-,09	-34*	-,17	,01	-,02	-,32*	-,09	,37**	,54**	,32*	,32*	,51**	1			
4a Attachment to internal objects	-,05	-,16	-,09	-,13	-,06	,02	,006	,32*	,58**	,38**	,51**	,41**	,49**	1		
4b Attachment to external objects	-,09	-,15	-,01	-,22	-,10	-,15	-,07	-,21	,31*	,26	,26	,48**	,35**	,55**	1	
5 Total structure	,01	-,16	-,14	,03	,08	-,09	,04	,43**	,65**	,44**	,42**	,59**	,65**	,58**	,53**	1

In the multivariate analysis, the other axes or sociodemographic data do not explain the main conflict and period of the disease. The sociodemographic variables influence the overall severity of violence those being age, income, and educational level of the victim and partner (F = 2.41, p <0.05), the severity of the other items of Axis I (F = 9.66, p <0.05), and by the existence of psychopathology–Axis V (F = 3.71, p <0.05). And the level of total structure was influenced by Axis V (F = 3.22, p <0.05) and by the items of Axis II: "Staying in the relationship and letting others act autonomously" and "Controlling, making orders and demands" from perspective B to the others (F = 9.42, p <0.001).

## Discussion

This cross-sectional study with fifty-six women victims of domestic violence from the South of Brazil showed that age, educational level, and income are determinants for some aspects of IPV against women. It was possible to build a psychodynamic diagnosis of them using the OPD-2 evaluation. They presented a median level of psychic structure, with conflict centered on the possibility of receiving care in exchange for tending to others and submissive in interpersonal relationships as a defense mechanism. 78.6% of the cases had some psychiatric disorder, like Major Depressive Disorder and Posttraumatic Stress Disorder.

### Comparison with other studies

Our findings are consistent with a Chilean study using OPD-2. Only one study on the applicability of OPD in the context of violence was found, which was a Chilean study with a sample of 28 women from a domestic violence care center in Santiago. The women that demonstrated the greatest severity of violence were those who reported a higher presence of depressive symptoms, PTSD, and lower educational level. Complementary, the main conflict prevailed: the “need for x self-sufficiency” (39%), followed by “submission x control” (50%) that may be related to revictimization. As well, the overall functioning of women victims of sexual violence is worse than other victims, since they suffer psychological and physical violence simultaneously, resulting in an accumulation of multiple traumas. The author still estimates that with the interruption of violence with the recovery process of the patient, these women will be able to recover their internal resources, since the psychic structure is a dynamic organization. Thus, the vulnerable structure due to the trauma suffered is considered an obstacle for the victims to manage their emotions and stress [12]. In this sense, there is an association between trauma experienced in childhood and occurrence of domestic violence [17, 18], since these subjects establish a bond of dependence with the other, in which the other takes advantage of this to a point where it becomes painful and degrading [19]. In this study, 57.1% of women reported not having witnessed discussions between parents during childhood when asked to mark the option "yes" or "no". A limitation with this process was women recognizing this issue, blaming only the partner and not associating it with the repetition of parental models. During the interview they described situations of childhood violence. This data was observed in the psychic structure (S1 Table) in which insecure internal objects were identified, difficulties in emotional regulation, perception of reality in a distorted way, and difficulties in recognizing their limitations and needs.

According to Caligor, Kernberg, and Clarkin [20], conflicts—conflicting motivations or impulses—are internalized patterns of relationships kept out of consciousness by defensive mechanisms, protecting the individual from threatening and painful aspects. In the case of these women, the main conflict "need for care x self-sufficiency" is in conformity with the literature, whose influence of social norms reinforces the submissive behavior of women in the face of coercive behavior of man [21]. However, studies show that women become submissive as a defense mechanism since they are afraid to suffer violence if they don’t act accordingly.

The dynamics of domestic violence implies a repetitive behavior pattern in relationships. The male perspective is characterized by abusive and coercive physical or non-physical conduct, with a recognizable inequality of powers and a set of forces. And concerning the female behavior, there is a presence of fear with a response of avoidance, adaptation, and submission. The dynamics established between the couple are cyclical: accumulation of tensions, conflict, and reconciliation phase [18, 22].

The structural axis—Axis IV—evaluates the level of integration of the patient's abilities or limitations in the regulation of mental functions capable of establishing internal homeostasis in the last two years. These functions integrate with capacities of the self, in the regulation of their internal experience, management of overload, and stress, allowing elaboration and adaptation. It is the result of a process of maturation and the development of internal representations of the world [8]. Their understanding integrates early attachment patterns from the internalization of mental representations [9]. In traumatic situations, evaluated in this axis, show deficits in mentalization capacity. In traumatic situations like violence in general, there may be the establishment of disorganized psychic structures. The division of object representations, instability in relationships, difficulty in the organization of a sense of identity, and failures in the capacity for mentalization/reflective function characterize psychological functioning [23]. Therefore, there are ruptures in the ability to think and reflect on the mental states of oneself and others [24].

Given this, victims list external causes to justify the IPV since their structural capacities are medium. The risk factors listed in the literature are related to the perpetrator are temperamental attitude, substance abuse like alcohol, witnessing family violence, and gender ideologies [18, 25].

Literature is controversial in some aspects of sociodemographic characteristics. Our research did not correspond to the sociodemographic data presented in literature [26, 27]; the crossing of class, race, and ethnic; showed that black women with lower educational level with poor living conditions were the main victims of violence and homicide. However, it corresponds to some of the aspects pointed out by the study Aziz et al. [25] regarding resources such as education, earnings, employment, and positive attitude towards beating women.

### Psychiatric diagnosis: Battered woman syndrome

78.6% of the cases suffered from some psychiatric disorder: Major Depressive Disorder (41.1%), Posttraumatic Stress Disorder (19.9%), and so forth. On these diagnoses, the research Lenore Walker created the “Battered Woman Syndrome” (BWS) [18]. The term appeared as a subcategory of Posttraumatic Stress Disorder. BWS consists of a pattern of signs and symptoms after a woman had an intimate relationship when the partner exercised power and control over the woman. Seven factors present in this study identify BWS, a) intrusive recollections of traumatic events, b) high levels of arousal and anxiety, c) avoidance behavior and emotional numbing usually expressed as depression, denial, minimization, and dissociation, d) disruption in interpersonal relationships of partner power.

### Strengths and limitations

We designed this study to reduce bias and provide aspects linking IPV against women. Firstly, we used the OPD-2 in the context of violence in Brazil, especially with the OPD Domestic Violence Assessment Module, the agreement between the judges was substantial. A limitation in some interviews was a coefficient between the judges lower than 0.60; however, we decided not to repeat the codification in order not to condition the evaluation of the items or to infer a certain interpretation on the judges.

Another strength of our study was the exploration of operationalized psychodynamic diagnosis of women victims of domestic violence, exploring the severity and experience of violence, structural functions, dysfunctional interpersonal patterns, and intrapsychic conflicts. Researchers point out that trauma, due to constant violence, causes changes in structural functioning and conflict [8].

Finally, our study population was representative of all social characteristics of Southern Brazil, which makes the findings generalizable. In numerous countries, IPV is common in many cases. Romagnoli [28] points out that this is a social and public health problem since it generates a high burden on the health system—calculated in terms of mortality/morbidity, quality of life, and cost; primarily affects minorities and disadvantaged individuals. Despite the protection and reception services, women remain in the cycle of violence, either with the same partner or with another, repeating the same behavioral patterns. It is important to identify the characteristics of the psychological functioning of these women to understand how and when they will break the cycle of victimization. However, this study did not intend to trace a single profile of women victims of violence, on the contrary, presenting different epidemiological characteristics.

Brazil faces several barriers in the Brazilian context since there is a social permissiveness in relation to aggression with the banalization of violent behavior against women. That is, it is naturalized in society [28, 29]. The culture of patriarchy is present, which was culturally created that male honor allows women to be beaten, threatened, and killed. In view of this, Law 11.340/06, known as Maria da Penha was created, which integrates principles regarding violence against women or gender violence. It creates legal mechanisms to prevent and punish domestic violence against women, that is domestic violence ceases to be a crime of minor offense to a criminal level of human rights violation [16].

### Implications for clinical practice and public health

According to Falcke and Féres-Carneiro [30] and Walker [18], women who suffer domestic violence are not easy to identify, since they hide the signs due to shame, but there is an immense change in personality. This violence reveals imprisonment in themselves, as they lose interest in social activities in preference to staying in the home environment and even naturalize violent behavior. Socially, there is the massive presence of the patriarchal ideology in the world, whose violence is associated with masculinity. The authors discuss the dynamics of partner violence since partner selection is a reproduction of violent behavioral patterns experienced in childhood and women's inability to reflect on their relationship choices due to traumatic events and lack of meaningful emotional experiences [31].

Victims of violence are considered hard patients because of their countertransference feelings of frustration [11]. Also, a source of psychic suffering for the therapist is treating traumatized patients since they have an intense and significant emotional load [32]. Therefore, a specific evaluation of the patient's violent context is necessary; identifying the patient's resources and obstacles; the personal explanation for victimization; the possible secondary gain, that is, investigating the characteristics and the psychodynamic functioning; and clinical understanding of these patients; the dimension axes of the OPD-2 can perform this analysis. During the psychotherapeutic process, stimulating the event through reflection and mentalization can trigger a traumatic reenactment. This can provide a new understanding of the violence experienced, producing new representations [33].

Regarding the individual resources, some characteristics help in the elaboration of the traumatic event, such as resilience capacity, the phase of life that the event occurred, and the previous history. The internalization of references to good relationships in childhood aid in the structuring of safer and more stable subjects. After a stressful occurrence, there may be the development of hyperstimulation, where anxiety is prevalent or dissociation, in which the individual seems to be numbed, but hides great inner suffering [34].

Due to violence, there is a change in the capacity of the self to handle the internal experience and the interaction with others; they have negative attitudes, shame, stigma, and difficulties in regulating their emotions in relationships with others [35]. For Johnson and Benight [36], the ability to tolerate trauma, due to domestic violence, refers to Bandura's self-efficacy; since, according to Campos, Faria, Zanini, and Peixoto [37], self-efficacy interferes with the individual's ability to produce certain results, overcoming difficulties and presenting control over the environment. And for Kane et al. [38], victims of trauma due to domestic violence who had greater social contact showed less effect in daily activities such as cooking, caring for family members, and work. Thus, aspects that interfere in the relationship between traumatization and illness, social support, social protection network, type of victimization, age, gender, perceived self-efficacy, and so forth [39]. Low levels of social support are related to higher rates of IPV [40]. Also, there are side effects on child care, mothers who have greater difficulties in showing affection to their child's needs, and some mother-child relationships become more tense and distant than others in domestic violence contexts [22].

Scientific evidence points to the association between domestic violence and mental health problems, such as depression, anxiety, suicide attempts, posttraumatic stress symptoms [1], as pointed out in this study. The results of the study by Schultz and co-workers [40] point to broad implications for IPV survivors in addition to increased depression, health conditions associated with type 2 diabetes (cardiovascular, ocular, urinary and circulatory) and drug abuse, quality of life in this population. Thus, the dimensional diagnosis complements the nosological diagnosis, which consequently facilitates the clinic.

## Conclusion

This study built empirical evidence on clinical observations regarding the psychological functioning of this population and the issues that make up the maintenance of domestic violence against women. The dynamics of victimization are hard to identify because victims are embarrassed to report or are not critical about the problem. The understanding of internalized patterns (Axis II), structural functions (Table 5), and motivational tensions (Fig 1) are fundamental for the prevention of re-victimization, and the construction of more adaptive coping mechanisms, as well as to promote greater adherence to treatment. The psychodynamic dimensional identification is a useful tool for therapeutic planning and focus of the demands that constitute the obstacles and impasses of the interruption of the cycle of violence.

It is noteworthy that women suffered very difficult situations and mentioned that they did not react out of fear or because they felt unable to do so (Table 1). They put themselves in a submissive position (Fig 1) as a way to anticipate the partners’ reaction. They perceive others as controlling and aggressive and, as a consequence, victims are insufficiently protected, allowing dangerous progression. They feel confused when the other shows affection, holding on and being dependent on the other. (Axis II). They do not recognize their limitations and needs due to IPV. Few perceive the similarity of repetition of parental patterns of violence. The situations and events that generate tension weaken the coherence of the self-image (Table 5). The victims believe that external measures were needed to overcome the cycle of violence, such as the protective measures provided by the protection agencies in the court (Table 3). Income was not a very significant factor in maintaining the cycle of violence, but the level of education influenced their perception and resources to deal with the situation of violence (Table 8).

### Unanswered questions and future research

Intimate partner violence against women is a cyclical phenomenon, so to conduct a cohort study to observe the particularities of victimization using a large sample and the OPD-2 to plan treatment would be interesting. Studies based on long-period follow-ups would make it possible to evaluate the evolution of the psychological functioning of women victims of IPV, especially after violence has ended or after the therapeutic process.

Future research should also include the effects of IPV on child-rearing since studies are pointing to the phenomenon of transgenerationality. As well as, investigate the influence of social support and specialized services, as well as the ability to soften the effects of traumatic stressors on health outcomes.

## Supporting information

S1 Table OPD-2 coding worksheet. (PDF)
