# Supplementary material for: Intimate partner violence against women: Operationalized Psychodynamic Diagnosis (OPD-2)
Source: PLoS One. 2020 Oct 1;15(10):e0239708. doi: 10.1371/journal.pone.0239708 (PMC7529253; doi:10.1371/journal.pone.0239708)
Supplement: S1 Table — (PDF) [file pone.0239708.s001.pdf]

Entrevista: ________________________________ **Data da avaliação: ___/___/_____**

Planilha de registro e codificação: Módulo para a Avaliação de Violência Doméstica OPD

Avaliação das características da violência doméstica por parte do clínico

1.- Tipo e severidade da violência

Emocional	⓪	①	②	③	④	⑨
Física	⓪	①	②	③	④	⑨
Sexual	⓪	①	②	③	④	⑨
Índice de severidade global	⓪	①	②	③	④	⑨

2. Duração da situação de violência doméstica

2.1. Duração da violência do parceiro	< 6 Meses	6-24 Meses	2-5 anos	5-10 anos	> 10 anos	⑨
2.2. Idade do primeiro episódio	Em anos ➔ ____					⑨

3.- Vivência subjetiva do problema por parte da paciente

**3.1- Intensidade do sofrimento subjetivo**	⓪	①	②	③	④	⑨
**3.2.- Apresentação de queixas sobre violência doméstica**	⓪	①	②	③	④	⑨

4- Conceito pessoal da violência doméstica

4.1.-Orientada para fatores externos	⓪	①	②	③	④	⑨
4.2.- Orientada para fatores psicológicos	⓪	①	②	③	④	⑨

5. – Conceito de mudança

5.1.- Orientado para mudanças ou medidas externas	⓪	①	②	③	④	⑨
5.2.- Orientado para aspectos pessoais	⓪	①	②	③	④	⑨

6 – Mudanças: recursos e obstáculos

6.1.- Recursos internos	⓪	①	②	③	④	⑨
6.2.- Obstáculos internos	⓪	①	②	③	④	⑨
6.3.-Recursos externos	⓪	①	②	③	④	⑨
6.4.- Obstáculos externos	⓪	①	②	③	④	⑨

Diagnóstico Psicodinâmico Operacionalizado 2: Formulários de avaliação de dados (KRIEGER, 2013)

Eixo I – Vivência da doença e pré-requisitos para o tratamento

	**Eixo I – Vivência da doença e pré-requisitos para o tratamento Módulo Básico**	**Nada/ Raramente presente**		**Moderado**		**Elevado**	**Não classificável**
		⓪	①	②	③	④	⑨

Avaliação objetiva da doença/problema

**1. Gravidade atual da doença/problema**						
1.1. Gravidade dos sintomas	⓪	①	②	③	④	⑨
1.2. GAF: máximo nos últimos 7 dias ➔ ______						⑨
1.3. EQ-5D: ________ Valores dos itens ➔	1.___	2.___	3.___	4.___	5.___	⑨
**2. Duração da doença/problema**						
2.1. Duração da doença	< 6 Meses	6-24 Meses	2-5 anos	5-10 anos	> 10 anos	⑨
2.2. Idade na primeira manifestação da doença	Em anos ➔ ____					⑨

Vivência, forma de apresentação e conceptualização da doença por parte do paciente

**3. Vivência e forma de apresentação da doença**						
3.1. Sofrimento subjetivo	⓪	①	②	③	④	⑨
3.2. Presença de problemas e queixas físicas	⓪	①	②	③	④	⑨
3.3. Presença de problemas e queixas psicológicos	⓪	①	②	③	④	⑨
3.4 Presença de problemas sociais	⓪	①	②	③	④	⑨
**4. Conceptualização o da doença por parte do paciente**						
4.1. Concepção da doença baseada em fatores somáticos	⓪	①	②	③	④	⑨
4.2 Concepção da doença baseada em fatores psicológicos	⓪	①	②	③	④	⑨
4.3. Concepção da doença baseada em fatores sociais	⓪	①	②	③	④	⑨
**5. Conceptualização da mudança por parte do paciente**						
5.1. tipo de tratamento desejado: físico/médico	⓪	①	②	③	④	⑨
5.2. tipo de tratamento desejado: psicoterapêutico	⓪	①	②	③	④	⑨
5.3. tipo de tratamento desejado: apoio social	⓪	①	②	③	④	⑨
**6. Recursos para a mudança (últimos 6 meses)**						
6.1. Recursos pessoais	⓪	①	②	③	④	⑨
6.2. Apoio (Psico)Social	⓪	①	②	③	④	⑨

7. Resistências à mudança						
7.1. Resistências externas à mudança	⓪	①	②	③	④	⑨
7.2. Resistências internos à mudança	⓪	①	②	③	④	⑨

Eixo II – Relações Interpessoais

**Perspectiva A: Vivência por parte do paciente**	
O paciente percepciona-se a si próprio como...	O paciente percepciona os outros como...
1. ______	1. ______
2. ______	2. ______
3. ______	3. ______
**Perspectiva B: A percepção dos outros (incluindo a do investigador)**	
Os outros percepcionam o paciente como...	Os outros percepcionam-se a si próprio como...
1. ______	1. ______
2. ______	2. ______
3. ______	3. ______

Eixo III – Conflito

A) Os conflitos não podem ser classificados por falta de segurança diagnóstica.	sim =①	não=⓪
B) Devido a um baixo nível de integração estrutural, não se reconhece um padrão distinto conflitual, mas antes padrões conflituais tênues.	sim=①	não=⓪
C) Uma vez que a percepção dos conflitos e dos afetos está condicionada por questões defensivas, o eixo do conflito não pode ser classificado.	sim =①	não=⓪
D) Stress conflitual (conflito indutor de stress) sem nenhum padrão conflitual e disfuncional repetitivo.	sim =①	não=⓪

**Conflitos disfuncionais repetitivos**	**Ausente**	**Insignificante**	**Significativo**	**Muito significativo**	**Não classificável**
1. Individuação versus Dependência	⓪	①	②	③	⑨
2. Submissão versus Controle	⓪	①	②	③	⑨
3. Necessidade de ser cuidado versus auto-suficiência	⓪	①	②	③	⑨
4. Conflito de Auto-estima	⓪	①	②	③	⑨
5. Conflito de culpa	⓪	①	②	③	⑨
6. Conflito edipiano	⓪	①	②	③	⑨
7. Conflito de identidade	⓪	①	②	③	⑨

Conflito principal: __________ Seguido de (por ordem de importância): __________

Modo como o conflito principal se processa	**Predominantemente activo**	**Misto mais activo**	**Misto mais pasivo**	**Predominantemente pasivo**	**Não classificável**
	①	②	③	④	⑨

Eixo IV – Estrutura

	Nivel Alto	1,5	Nivel Medio	2,5	Nivel Baixo	3,5	Nivel desinte-grado	Não classificável
1a Auto-percepção	①		②		③		④	⑨
1b Percepção do objeto	①		②		③		④	⑨
2a Auto-regulação	①		②		③		④	⑨
2b Regulação da relação objetal	①		②		③		④	⑨
3a Comunicação Interna	①		②		③		④	⑨
3b Comunicação com o mundo externo	①		②		③		④	⑨
4a Capacidade de vinculação: objetos internos	①		②		③		④	⑨
4b Capacidade de vinculação: objetos externos	①		②		③		④	⑨
**5** Estrutura total	①		②		③		④	⑨

Eixo V – Transtornos mentais e psicossomáticos

**Va: transtornos mentais:**	**CID-10** (criterios de investigación)	**DSM-IV** (opcional)
Diagnóstico principal:	**F _____. _____**	______. ____
